# Ovarian Cancer: From Precursor Lesion Identification to Population-Based Prevention Programs

**DOI:** 10.3390/curroncol30120741

**Published:** 2023-11-29

**Authors:** Ramlogan Sowamber, Alexandra Lukey, David Huntsman, Gillian Hanley

**Affiliations:** 1 Department of Gynecology and Obstetrics, University of British Columbia, Vancouver, BC V6T 1Z4, Canada; rsowamber@bccrc.ca; 2 Department of Medical Genetics, University of British Columbia, Vancouver, BC V6T 1Z4, Canada; 3 Department of Molecular Oncology, British Columbia Cancer Research Centre, Vancouver, BC V6T 1Z4, Canada; 4 School of Population and Public Health, University of British Columbia, Vancouver, BC V6T 1Z4, Canada; alex.lukey@ubc.ca; 5 Department of Pathology and Laboratory Medicine, University of British Columbia, Vancouver, BC V6T 1Z4, Canada

**Keywords:** ovarian cancer, cancer prevention, salpingectomy, opportunistic salpingectomy, fallopian tube

## Abstract

Epithelial ovarian cancer (EOC) is a heterogeneous group of malignancies, including high-grade serous ovarian cancer (HGSC). HGSC is often diagnosed at advanced stages and is linked to TP53 variants. While *BRCA* variants elevate risk, most HGSC cases occur in individuals without known genetic variants, necessitating prevention strategies for people without known high-risk genetic variants. Effective prevention programs are also needed due to the lack of traditional screening options. An emerging primary prevention strategy is opportunistic salpingectomy, which involves removing fallopian tubes during another planned pelvic surgery. Opportunistic salpingectomy offers a safe and cost-effective preventative option that is gaining global adoption. With the publication of the first cohort study of patients who underwent salpingectomy, specifically for cancer prevention, attention has turned to broadening opportunities for salpingectomy in addition to more targeted approaches. Prevention opportunities are promising with increasing adoption of salpingectomy and the increased understanding of the etiology of the distinct histotypes of ovarian cancer. Yet, further research on targeted risk-reducing salpingectomy with thoughtful consideration of equity is necessary to reduce death and suffering from ovarian cancer.

## 1. Introduction

Epithelial ovarian cancer (EOC) or ovarian carcinomas, which are the fifth leading cause of cancer deaths in females [1], are heterogeneous malignancies consisting of five histotypes: low grade serous carcinoma (LGSC), mucinous carcinoma (MC), clear cell ovarian carcinoma (CCOC), endometrioid ovarian carcinoma (ENOC), and high-grade serous ovarian cancer (HGSC). As each histotype features unique genomic profiles, which translates into specific clinical behaviors, they are considered distinct diseases [2]. HGSC is the most lethal gynecological malignancy and accounts for up to 70% of epithelial ovarian cancers [3]. HGSC is characterized by genomic instability and TP53 variants, all contributing to a poor five-year overall survival rate [4]. Histologically, HGSC appears poorly differentiated with profound nuclear atypia and strong p53 and Ki67 staining; many cases have multinucleated tumor cells [5,6,7,8]. Individuals with *BRCA1/2* variants have an increased risk of developing HGSC [9,10,11]. Calculated to age 80, *BRCA1* variant carriers have a cumulative risk of ovarian cancer of 36%–53%, and carriers of the *BRCA2* variant have a cumulative risk of 11%–25% [12]. In a cohort of 367 individuals, 87 individuals presented with 88 mutations in germline homologous recombination genes, with 49/88 mutations (54%) occurring in *BRCA1* and 17/88 (19%) in *BRCA2* [13]. Moreover, 35 loss-of-function somatic mutations were identified in 32/367 individuals, of which 19/35 (54%) mutations were in *BRCA1,* and six (17%) were in *BRCA2*, which was of consideration for selecting patients for treatment with poly (ADP-ribose) inhibitor (PARPi) [13]. More recently, however, homologous recombination deficiency (HRD) testing, which is a Food and Drug Administration (FDA) approved method of selecting ovarian cancer patients for PARPi treatment, has demonstrated a significant benefit to ovarian cancer patients by using HRD status to predict overall survival and progression free survival of patients undergoing PARPi treatment [14,15,16,17]. Despite a dramatically increased risk among people with a *BRCA* variant, more than 80% of high-grade serous ovarian cancers arise in people with no known genetic variants [18,19]. Thus, prevention strategies need to be aimed at the general population as well as high-risk populations.

Ovarian cancer is most often diagnosed at later stages (Stage III/IV). Screening methods are ineffective in reducing ovarian cancer deaths, as evidenced by the UKCTOCS screening trial in 2021 [20]. Despite improved treatment options and a reduction in incidence due to a hypothesized link to the widespread use of oral contraceptives, ovarian cancer has the highest mortality rate of gynecologic malignancies [21,22,23,24]. Furthermore, most people diagnosed with ovarian cancer will initially respond to chemotherapy and then recur with resistant disease [25,26]. Thus, a focus on prevention makes a lot of sense for ovarian cancer. Along with the realization that the fallopian tube is the tissue of origin for most high-grade serous cancer (outlined in detail below), an important primary prevention strategy was introduced in 2010 in British Columbia, Canada, called opportunistic salpingectomy, which is the removal of the fallopian tubes during other pelvic surgery while leaving the ovaries intact [27]. Since then, it has gained traction amongst the gynecological community. Here, we aim to review updated evidence related to the origins of ovarian cancer and current prevention strategies and discuss future considerations for ovarian cancer prevention.

## 2. Expanding the Understanding of the Origins of Ovarian Cancer

### 2.1. Origins of High-Grade Serous Ovarian Cancer

For almost a century, it was believed that ovarian cancer derived from the ovarian surface epithelium [28]. This view of the origin of HGSC was challenged in 1999 in an editorial and then shifted two years later when a group of researchers from the Netherlands discovered early pre-cancerous lesions in the fallopian tubes of *BRCA* variant carriers, called serous tubal intraepithelial carcinomas (STICs) [29,30]. In 2006, the introduction of the Sectioning and Extensively Examining the Fimbriated End (SEE-FIM) protocol standardized the pathological review procedure of fallopian tubes, including the fimbria, and highlighted the importance of the fimbriated end as a site of origin for HGSC [31]. One study showed the presence of four tumors in the fimbriated end and one tumor in the ampulla in a cohort of 26 prophylactic bilateral salpingo-oophorectomy cases, highlighting the benefits of the SEE-FIM protocol for review of precancerous lesions [31,32]. The origin of HGSC from the fallopian tube occurs with the accrual of genetic aberrations in normal fallopian tube followed by a p53 signature [33,34]. Support, given through a histological review of 75 fallopian tubes from *BRCA* variant carriers, demonstrated p53 signatures in 38% of fallopian tubes [35] with mutant pattern nuclear p53 staining, mild Mib1 (Ki67) staining of 0–30%, and the presence of yH2AX foci, which indicates increased DNA damage [36]. The p53 signature is a strip of 10–30 fallopian tube epithelial cells characterized immunohistochemically through positive p53 protein expression [6,37]. Not all p53 signatures progress to a STIC and HGSC. One model of early precursor lesion development shows that it takes a few decades for p53 signatures to progress into a STIC, but only six to seven years for a STIC to transition into HGSC [38]. STICs are precancerous lesions that present with identical genomic aberrations to HGSC [39]. A review of 29 pelvic HGSCs showed identical somatic p53 variants in 27 concurrent STIC and HGSC pairs, with 61% of STIC cases having missense variants [34]. STICs are characterized by nuclear atypia, p53 staining, increased proliferation, and loss of cell polarization. The diagnosis and inter/intra-reproducibility of STICs varies between studies; nonetheless, the general trend suggests these lesions are less frequently observed in individuals from the general population who do not have a *BRCA* variant [32,40,41,42,43,44,45]. One study of 176 *BRCA* variant carriers and 64 controls found tubal intraepithelial carcinoma in 8% of *BRCA* variant carriers and 3% of controls [46]. The co-existence of STICS and HGSC in individuals without a *BRCA* variant was 33% in one study [47] and 66% in another study [48] and ranged from 11% to 61% based on a meta-analysis of studies with the inclusion of STICs and HGSCs [49]. The presence or absence of STIC lesions did not show significant differences in copy number alterations and RNA profiles [7]. There is now substantial evidence that supports the fallopian tube secretory epithelial cell as the cell of origin for HGSC [33,50,51].

**Figure 1:**
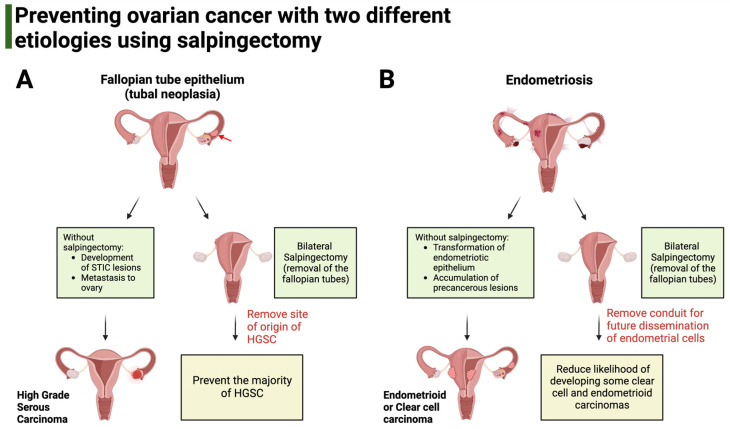
Salpingectomy aims to prevent ovarian cancers with different etiologies. (**A**). HGSC derives from genetic alterations of the fallopian tube epithelium that accumulate in the form of neoplasia (red arrow). Without salpingectomy, precancerous lesions called serous tubal intraepithelial carcinoma continue to develop, eventually giving rise to HGSC. Salpingectomy aims to prevent HGSC by removing the anatomical site of origin for these cancers. (**B**). The origin of clear cell carcinoma and endometrioid carcinoma is endometriosis. Bilateral salpingectomy aims to prevent these two carcinomas by removing the fallopian tube, which can act as a conduit for the dissemination of altered endometrial cells to the ovary and peritoneal cavity.

In addition to the fallopian tube epithelium, other ovarian cancers, like clear cell carcinoma and endometrioid carcinoma, can arise from endometriosis (ectopic endometrium) and possibly from endosalpingiosis (ectopic fallopian tube epithelium), both of which are thought to use the fallopian tube as a conduit for dissemination to other structures of the peritoneal cavity (Figure 1) [52,53].

The tubal dissemination hypothesis suggests STIC lesions detach from the fallopian tube epithelium and migrate onto the ovary and into the peritoneal cavity for implantation [54]. Subsequent genomic alterations and natural selection allow the STIC to survive and grow in new environments, including the closely positioned ovary [55]. Compared to normal fallopian tube epithelium, STIC lesions have significantly more somatic variants and loss of heterozygosity (LOH) alterations [56]. Some patients that harbor TP53 signatures and STICs with no corresponding HGSC tumors have shorter telomeres compared to normal epithelium [57]. A subset of STICs with corresponding HGSC showed the shortest telomere lengths compared to normal epithelium and other STICs without a HGSC [57]. Together, this knowledge demonstrates the presence of a precursor lesion within the fallopian tube epithelium, giving rise to HGSC. However, clonal evolutionary analysis shows that a proportion of STIC lesions could be intraepithelial metastases that are not clonally related to HGSC. This suggests there may be an alternative developmental pathway for a subset of HGSCs [28,58].

### 2.2. The Fallopian Tube as a Conduit for Genomically Unstable Cells

The involvement of the fallopian tube in the pathogenesis of other ovarian cancers like clear cell carcinoma and endometrioid carcinoma is indirect as compared to HGSC. Patients with endometriosis are at a 2–3-fold increased risk for clear cell carcinoma and endometrioid ovarian carcinoma, cancers that are commonly referred to as endometriosis-associated ovarian cancer [59,60,61,62,63]. Endometriosis is a common chronic inflammatory disease affecting ~10% of women and is characterized by lesions resembling the uterine endometrium found in the abdomen at locations outside the uterus, primarily elsewhere in the pelvis [64]. It is hypothesized that retrograde menstruation is an important source of endometriosis [65,66]. The fallopian tube involvement in endometriosis is less well studied, but there are accounts of endometriosis following tubal sterilization [67,68]. In addition, the fallopian tube acts as a conduit for the dissemination of genomically altered cells to the peritoneal cavity. Exome sequencing of lesions from deep infiltrating endometriosis shows somatic variants in oncogenic driver genes such as ARID1A, PIK3CA, KRAS, or PP2R1A [69]. 

Endometriosis-associated endometrioid carcinoma (EAEOC) is associated with younger age at diagnosis and lower disease stage compared to individuals with endometrioid carcinoma not associated with endometriosis [70,71]. Individuals with ovarian clear cell carcinoma present symptoms during middle to older age (mean age = 56 years old) [72] and have lower response to anticancer drugs, [73] with pure clear cell carcinomas having worse prognosis compared to admixed clear cell carcinoma [74,75]. Clear cell carcinomas frequently present with variants in ARID1A and PIK3CA, which have been found in adjacent endometriosis variants [76,77,78]. Endometrioid ovarian carcinomas are essentially endometrial carcinomas in the wrong place, and the same molecularly defined prognostic subgroups can be seen in both diseases [79]. Significant genetic overlap exists between endometriosis and epithelial ovarian cancers (HGSC, clear cell carcinoma, and endometrioid carcinoma) [61], with single-cell transcriptomic analysis highlighting an inflammatory, pro-angiogenic, and pro-lymphangiogenic environment in endometriosis cases [80]. There is epidemiologic evidence suggesting fallopian tube involvement with endometriosis-associated ovarian cancers. A pooled analysis found that tubal ligation was associated with an approximate 50% reduction in endometrioid and clear cell carcinomas [81].

**Figure 2:**
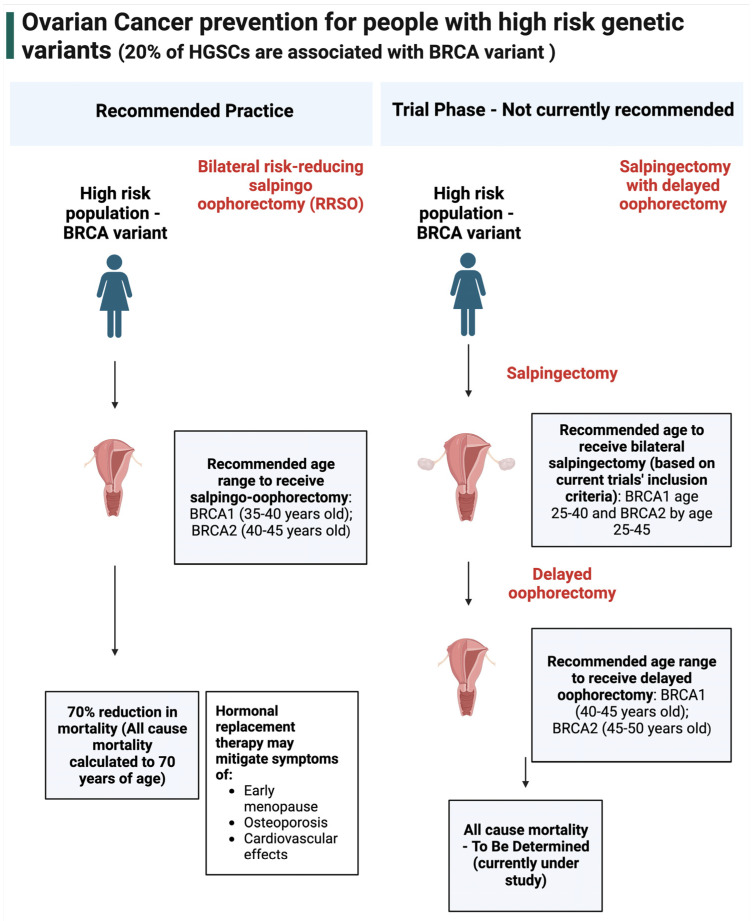
Ovarian cancer prevention for people with high−risk genetic variants. In high-risk populations, the current recommended method of prevention is risk reducing salpingo-oophorectomy, which is the removal of the fallopian tubes and ovaries [89]. This prevention method reduced all-cause mortality by up to 70% (after a follow-up period of 5.6 years, calculated to age 70) [90]. These individuals are provided with hormonal replacement therapy to mitigate the effects of oophorectomy, which includes early menopause, osteoporosis, and cardiovascular effects. Salpingectomy with delayed oophorectomy is currently in the trial phase and is not recommended for general practice.

Over the last 20 years, support for the fallopian tube as the origin of HGSCs has led to increased interest in salpingectomy. This surgical procedure removes the fallopian tubes while leaving the ovaries intact [82]. Current practice for high-risk individuals (BRCA variant carriers) is risk reducing bilateral salpingo-oophorectomy (RRBSO) (Figure 2) [83] with the recommendation of hormone therapy, provided there are no contraindications [84]. Ongoing clinical trials are testing whether salpingectomy followed by delayed oophorectomy in BRCA variant carriers could provide the same risk reduction of RRBSO but without the damaging effects of premature menopause (https://clinicaltrials.gov/, NCT02760849, NCT02321228, accessed 10 September 2023). However, for the general population, who represent 80% of ovarian HGSC cases that occur without a germline BRCA or equivalent variant, RRBSO is not recommended since it predisposes a female to early menopause, osteoporosis, and other health-related impacts [85]. More specifically, opportunistic salpingectomy, which is the removal of the fallopian tubes at the time of other surgical procedures such as a hysterectomy or tubal ligation, provides a direct means of preventing ovarian cancer in women without causing surgical menopause. Therefore, opportunistic salpingectomy has been recommended for individuals of the general population who do not desire a future pregnancy [86,87,88]. 

**Figure 3:**
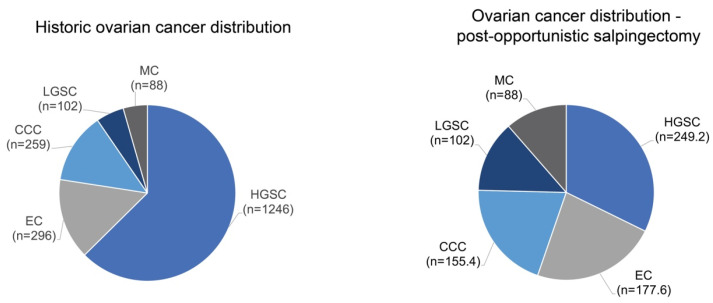
Projected histotype shift following salpingectomy. The historic ovarian cancer distribution for individuals from the general population shows that HGSC is the most common histotype. In those who have received salpingectomy, it is anticipated that the proportion of HGSC will decrease relative to other histotypes.

For people who have undergone salpingectomy, we predict a reduction in HGSC by 80% and clear cell and endometrioid carcinoma by 40% [18]. The historic ovarian cancer distribution from a 2018 Canadian Ovarian Experimental Unified Resource (COEUR) cohort, which was reclassified using updated histological classification, shows that for people in the general population who undergo salpingectomy, there would be a projected reduction of 80% for HGSC (Figure 3) [91]. 

## 3. Current Evidence for Opportunistic Salpingectomy

### 3.1. Screening

The need for ovarian cancer prevention efforts is particularly relevant, with recently published evidence highlighting the lack of options for population screening. Screening is a pillar of cancer prevention. However, for screening to be worthwhile, several conditions must be met. There must be reasonable disease prevalence, the disease must be an important cause of mortality, have a detectable preclinical phase, and be treatable [92]. While ovarian cancer is certainly an important cause of mortality, it has available treatments and is reasonably prevalent to warrant screening; current screening methods do not seem adequate to detect the preclinical phase. In the UKCTOCS trial, which aimed to test population screening to prevent ovarian cancer, the trial achieved a stage shift but unfortunately found no difference in deaths from ovarian cancer when using multimodal screening or annual transvaginal ultrasound compared to no screening [20]. This mortality result aligns with a previous review of four screening trials, of which none found a significant reduction in deaths from ovarian cancer [93]. Thus, there remains no effective screening method for ovarian cancer. 

### 3.2. Safety of Salpingectomy

Multiple publications have illustrated that opportunistic salpingectomy does not increase the risk of perioperative adverse outcomes [94,95], nor does it increase the risk of minor complications [96]. Further safety evidence is now available regarding OS during vaginal or caesarean delivery for people seeking permanent contraception. A systematic review and meta-analysis of salpingectomy at the time of cesarean delivery included 320,443 people and compared salpingectomy to standard sterilization methods and found no increase in wound infection, blood transfusion, readmission, reoperation, internal organ damage, blood loss, change in hemoglobin, or length of stay [97]. 

However, there remains some concern that salpingectomy may reduce the age of onset of menopause through disruption of blood flow to the ovaries. Now, further safety evidence is available regarding the risk of early menopause associated with salpingectomy. A systematic review and meta-analysis, which included 15 studies that examined the effect of salpingectomy on the ovarian reserve, found no differences in the measures of the ovarian reserve, including anti-Müllerian hormone, antral follicle count, estradiol, follicle stimulating hormone, and luteinizing hormone [98]. However, the ranges of anti-Mullerian hormone concentrations reported across studies suggested that the onset of menopause could occur somewhere between 0 and 20 months earlier in the hysterectomy with the salpingectomy group compared to a hysterectomy alone. Another population-based study found that of 4952 patients who underwent opportunistic salpingectomy, there were no significant differences in time to the first physician visit related to menopause or to fill a prescription for hormone replacement therapy [99]. However, this finding conflicted with a Swedish registry study reporting more menopausal symptoms among women who underwent a hysterectomy with opportunistic salpingectomy one year after the surgery [100]. Therefore, whereas most evidence suggests opportunistic salpingectomy is unlikely to result in any major changes to the age of onset of menopause, this remains an unsettled area of science. 

Additionally, the cost-effectiveness of opportunistic salpingectomy compared to tubal ligation immediately after vaginal delivery was modelled by Wagar et al., showing that opportunistic salpingectomy was more cost-effective than tubal ligation with an incremental cost-effectiveness ratio (ICER) of USD 26,150/QALY, showing potential cost savings of the procedure in cases where permanent contraception is desired [101]. Similarly, compared to tubal ligation at the time of caesarean delivery, opportunistic salpingectomy was shown to have an ICER of USD 23,189/QALY [102]. Furthermore, when comparing opportunistic salpingectomy at the time of hysterectomy for benign conditions compared to tubal ligation, opportunistic salpingectomy was found to be less costly to the health system in the long term compared to tubal ligation [103].

### 3.3. Effectiveness of Salpingectomy

#### 3.3.1. Historical Studies

Given the recency of the recommendation for opportunistic salpingectomy, the age at which most people get opportunistic salpingectomy, and the follow-up time needed to see an effect on ovarian cancer risk, most evidence supporting opportunistic salpingectomy comes from studies of historical salpingectomy. This means patients were receiving salpingectomy for reasons other than ovarian cancer prevention. Even so, historical studies have consistently shown a decreased risk of ovarian cancer after tubal sterilization. The decreased risk conferred from salpingectomy was stronger (35%–64%) than non-excisional sterilization methods such as tubal ligation (13%–28%) [104,105,106]. Additionally, when comparing bilateral salpingectomy to unilateral salpingectomy, bilateral confers roughly twice the ovarian cancer risk reduction compared to unilateral salpingectomy [106,107]. Two recent case–control studies have been published that included data from both before and after opportunistic salpingectomy began to be recommended. One case–control study that identified 16,822 ovarian cancer cases, each matched with 40 controls from 1978–2019, found a 54% decrease in the odds of ovarian cancer for those that received bilateral salpingectomy (OR = 0.46; 95% CI: 0.31–0.67) [107]. Another recent case–control study including women from 1992–2019 found that cases that received bilateral salpingectomy had a nonsignificant 45% decrease in the risk of ovarian cancer compared to a nonsurgical control cohort [108]. Although, as the authors stated, the analysis was underpowered to detect a significant difference.

#### 3.3.2. Studies of Opportunistic Salpingectomy Performed for Ovarian Cancer Prevention

In a population-based retrospective cohort of 25,889 patients who underwent OS, there were no serous ovarian cancers and five or fewer epithelial ovarian cancers in the group that received opportunistic salpingectomy [18]. This was significantly lower than the group’s expected number of ovarian cancers [18]. There was also no significant difference in the number of breast or colorectal cancers in the opportunistic salpingectomy group, reducing the likelihood that the outcome of fewer ovarian cancers was due to unmeasured confounding factors. Overall, these results are promising, given their consistency with other epidemiologic data and our understanding of the development of HGSC [52,106]. 

### 3.4. Uptake in the General Risk Population

With the release of multiple national guidelines and increasing evidence for safety and effectiveness, opportunistic salpingectomy uptake is on the rise. In 2018, 13 of the 130 member countries of the International Federation of Gynecology and Obstetrics (FIGO) issued a statement on opportunistic salpingectomy. Of the thirteen countries, nine were supportive of opportunistic salpingectomy, four did not clearly recommend for or against opportunistic salpingectomy, and none advised against the procedure [109]. In Canada, between 2011 and 2016, the proportion of hysterectomies that included salpingectomy rose from 15.4% to 35.5% [110]. Opportunistic salpingectomy has been described as the new ‘de facto’ standard in Germany, as described by a survey of German gynecologists in 2022 [111]. In the survey of 166 gynecologists, 89% of gynecologists surveyed performed opportunistic salpingectomy in >50% of eligible cases in 2022. Furthermore, the number of salpingectomy cases reported in German public hospitals quadrupled between 2005 to 2022 [111]. In a retrospective study of Dutch hospitals, opportunistic salpingectomy performance increased from 6.9% in 2015 to 44.5% in 2018 in cases where patients underwent elective non-obstetric abdominal surgery for a gynecological indication [112]. From 2010 to 2017, age-adjusted incidence rates of opportunistic salpingectomy for sterilization or during hysterectomy increased 17.8-fold (95% CI: 16.2–19.5) and 7.6-fold (95% CI: 5.5–10.4) in a sample of 48,231,235 of inpatient and outpatient insurance claims from the United States [113].

## 4. The Power of Prevention: Strategies for Scaling Ovarian Cancer Prevention in the Future

### 4.1. Expanding Access

While opportunistic salpingectomy is clearly an important way forward for the primary prevention of ovarian cancer, if the goal is to reduce the incidence of ovarian cancer, a significant challenge will be ensuring there are enough ‘opportunities’ to conduct ‘opportunistic salpingectomies’. Many researchers are already exploring expanding salpingectomy to non-gynecological abdominopelvic procedures. Tomasch et al. proposed opportunistic salpingectomy during laparoscopic cholecystectomy, which has been shown to be cost-effective with an ICER ranging from USD 11,162 to 26,463 [114,115]. Qualitative data also supports that patients would accept being offered salpingectomy at the time of laparoscopic cholecystectomy [116]. In Canada, a trial is also underway that examines the safety and uptake of opportunistic salpingectomy at the time of colorectal surgery (https://clinicaltrials.gov/, NCT05300711, accessed on 15 August 2023). Stone et al. recommended taking opportunities, including cholecystectomy, hernia repair, appendectomy, and urological surgeries, as potential targets to increase salpingectomy uptake [117]. 

Researchers are also beginning to investigate targeted risk-reducing salpingectomies. We may get the greatest reduction in mortality and morbidity from high-grade serous ovarian cancer by offering salpingectomy to people at a higher-than-average lifetime risk for ovarian cancer, what we are calling ‘risk-reducing salpingectomy (RRS)’. While people with pathogenic variants that increase the risk for ovarian cancer, including *BRCA* variants, would continue to receive risk-reducing bilateral salpingo-oophorectomy for ovarian cancer prevention, many people at higher-than-average lifetime risk do not have any known pathogenic variants [118]. The next logical step in ovarian cancer prevention is to study the acceptability and effectiveness of risk-reducing salpingectomy for individuals at a significantly higher lifetime risk than 1.4% (the average lifetime risk for the general population), yet for whom oophorectomy may be overly aggressive given the detrimental hormonal consequences. 

There are a variety of reasons that people may be at a higher lifetime risk for ovarian cancer, including common susceptibility variants, which can explain about 6% of the heritability of ovarian cancer [119], as well as differences in known risk and protective factors. Other well-known protective factors for ovarian cancer include lifetime use of combined oral contraceptives, aspirin, parity, tubal ligation, and breastfeeding, which are protective, as well as factors that increase lifetime risk, such as menopausal hormone replacement therapy use, family history of ovarian cancer, and endometriosis [118]. These validated risk and protective factors have allowed for increased precision in estimating an individual’s lifetime risk of ovarian cancer. In fact, an ovarian cancer risk prediction tool is now publicly available and validated. The CanRisk tool has been used to provide information on risk for breast and ovarian cancer since 2007 and has been recommended for use by several national bodies [120]. The model includes known risk factors and has been validated for ovarian cancer risk assessment in an independent data set from a prospective trial. In the prospective cohort, the model predicted 391 cancers, compared to 374 observed cancers (E/O = 1.05, 95% CI: 0.94 to 1.16) [121]. Thus, as our understanding of the risk factors for ovarian cancer and ability to predict its risk improve, the future of ovarian cancer prevention may include targeting salpingectomy to prevent ovarian cancers in people at higher-than-average lifetime risk for HGSC.

A third area of interest is improving uptake during gynecologic surgeries. Evidence supports that opportunistic salpingectomy uptake is primarily provider driven. When properly counselled, eligible patients overwhelmingly choose to undergo salpingectomy, with rates as high as >95% [112]. However, while guidelines encourage gynecologists to counsel patients about salpingectomy if they are having pelvic surgery and do not desire a future pregnancy, there has not been significant direction or support for how counselling ought to be done, leading to wide variation in practice, although there is one patient decision aide available [122,123]. Supportive of this need, Gelderblom et al. found that both patients and professionals thought counselling materials would facilitate salpingectomy uptake [124]. 

### 4.2. Equity

So far, opportunistic salpingectomy uptake has been far from equitable between populations [125]. Across geographies, individuals in rural locations are less likely to undergo opportunistic salpingectomy than those in urban locations [113]. Differences in rates of alternative surgical approaches may play a role here and warrant investigation. Karia et al. also found significant inequities between racial and ethnic groups in the United States. In multivariate-adjusted models of 650,905 people, non-Hispanic Black, Hispanic, and non-Hispanic ‘Other’ people were less likely to receive opportunistic salpingectomy than non-Hispanic White people [126]. These results were reinforced with a retrospective study that found that, after controlling for confounders, Black patients were almost 50% less likely to receive opportunistic salpingectomy compared to tubal ligation when sterilization was performed at the time of caesarian section [127]. In this case, while the outcome of permanent contraception was completed, Black patients did not receive the full benefit of salpingectomy. 

The causes of inequity in opportunistic salpingectomy delivery are multifaceted. These inequities arise in part due to the history of injustices and violence, such as forced and coerced sterilization targeted at populations, including immigrants, Black, Latina, and Indigenous people; people with disabilities; and people with chronic medical conditions, such as epilepsy [128]. In Canada, reports of forced or coerced sterilization of Indigenous females have been reported as recently as 2018 [129]. Notably, groups often targeted for forced or coerced sterilization have also been denied equitable treatment and preventative health care [130]. Additionally, some policies, such as the Medicaid policy on sterilization, which was created to protect vulnerable populations, may limit the autonomy of those it was designed to protect [131]. A 2023 study showed that of patients who desired permanent contraception, those with Medicaid insurance were less likely to receive requested permanent contraception compared to those with private insurance [132]. Other likely precipitating factors of inequity in opportunistic salpingectomy delivery include provider bias, lack of insurance coverage, quality of healthcare institutions, and differing healthcare literacy levels [94,113,124,133]. If opportunistic salpingectomy is to be delivered equitably, researchers, clinicians, and policymakers must account for historical injustices while protecting patient autonomy, lest we risk entrenching health inequity by failing to offer preventative care to people who have been the targets of these injustices.

## 5. Conclusions

Increasing confidence in our understanding of the origins of ovarian cancer alongside the safety and effectiveness of salpingectomy as a prevention strategy for ovarian cancer has led to increased adoption of opportunistic salpingectomy in the medical community [18,98,109]. However, the current uptake of salpingectomy relies on patient and provider awareness, leading to significant variation in practice and risks entrenching existing health inequities [122,125]. Furthermore, the opportunities for salpingectomy are currently limited to gynecological abdominal surgeries. Immediate next steps to scale salpingectomy include increasing ‘opportunities’ for salpingectomy, such as extending safety evidence to other procedures and training general surgeons to complete salpingectomy, work that is currently in the trial stage, as well as further investigating the role that targeted risk-reducing salpingectomy might play. More work is also needed to ensure that providers are counselling all eligible patients about salpingectomy. This could include increasing education and awareness of salpingectomy and scaling up currently available decision-making tools [123].
